# Obestatin signalling counteracts glucocorticoid‐induced skeletal muscle atrophy via NEDD4/KLF15 axis

**DOI:** 10.1002/jcsm.12677

**Published:** 2021-03-09

**Authors:** Tania Cid‐Díaz, Saúl Leal‐López, Fátima Fernández‐Barreiro, Jessica González‐Sánchez, Icía Santos‐Zas, Luis J. Andrade‐Bulos, Manuel E. Rodríguez‐Fuentes, Carlos S. Mosteiro, Vincent Mouly, Xesús Casabiell, Jose Luis Relova, Yolanda Pazos, Jesus P. Camiña

**Affiliations:** ^1^ Laboratorio de Endocrinología Celular, Instituto de Investigación Sanitaria de Santiago (IDIS) Complejo Hospitalario Universitario de Santiago (CHUS), Servicio Gallego de Salud (SERGAS) Trav. Choupana s/n Santiago de Compostela Spain; ^2^ Laboratorio de Patología Digestiva, Instituto de Investigación Sanitaria de Santiago (IDIS) Complejo Hospitalario Universitario de Santiago (CHUS), Servicio Gallego de Salud (SERGAS) Trav. Choupana s/n Santiago de Compostela Spain; ^3^ Paris Cardiovascular Research Center‐PARCC Université Paris Descartes, Sorbonne Paris Cité, INSERM UMRS 970 Paris France; ^4^ Departamento de Fisiología Universidade de Santiago de Compostela (USC) Santiago de Compostela Spain; ^5^ Center of Research in Myology Sorbonne Universités, UPMC Univ Paris 06, INSERM UMRS 974 Paris France

**Keywords:** Skeletal muscle, Glucocorticoid‐induced muscle atrophy, Obestatin signalling, KLF15, NEDD4, FoxO

## Abstract

**Background:**

A therapeutic approach for the treatment of glucocorticoid‐induced skeletal muscle atrophy should be based on the knowledge of the molecular mechanisms determining the unbalance between anabolic and catabolic processes and how to re‐establish this balance. Here, we investigated whether the obestatin/GPR39 system, an autocrine signalling system acting on myogenesis and with anabolic effects on the skeletal muscle, could protect against chronic glucocorticoid‐induced muscle atrophy.

**Methods:**

In this study, we used an *in vivo* model of muscle atrophy induced by the synthetic glucocorticoid dexamethasone to examine the liaison molecules that define the interaction between the glucocorticoid receptor and the obestatin/GPR39 systems. The findings were extended to *in vitro* effects on human atrophy using human KM155C25 myotubes.

**Results:**

KLF15 and FoxO transcription factors were identified as direct targets of obestatin signalling in the control of proteostasis in skeletal muscle. The KLF15‐triggered gene expression program, including atrogenes and FoxOs, was regulated via KLF15 ubiquitination by the E3 ubiquitin ligase NEDD4. Additionally, a specific pattern of FoxO post‐translational modification, including FoxO4 phosphorylation by Akt pathway, was critical in the regulation of the ubiquitin–proteasome system. The functional cooperativity between Akt and NEDD4 in the regulation of FoxO and KLF15 provides integrated cues to counteract muscle proteostasis and re‐establish protein synthesis.

**Conclusions:**

The effective control of FoxO activity in response to glucocorticoid is critical to counteract muscle‐related pathologies. These results highlight the potential of the obestatin/GPR39 system to fine‐tune the effects of glucocorticoids on skeletal muscle wasting.

## Introduction

The actions of adrenal glucocorticoids (GCs) are mediated by glucocorticoid receptors (GRs), prototypic member of the nuclear receptor superfamily, which act as a ligand‐dependent transcription factor.^1^ Upon binding GCs, GRs translocate into the nucleus and bind to the glucocorticoid response element (GRE) in the promoters of target genes.^1^ The binding of liganded receptors to target DNA is followed by the recruitment of mediators and coactivators to the proximity of GRE, resulting in the recruitment of RNA polymerase II to adjacent transcription start sites and the activation of transcription.^1^, ^2^, ^3^ In skeletal muscle, GCs elicit a variety of biological actions in the metabolism of lipids, glucose, and proteins and contribute to metabolic homoeostasis.^4^ On the other hand, the prolonged over‐secretion or exogenous administration of GCs results in undesirable effects including muscle atrophy.^5^, ^6^ Although many studies addressed the mechanism of GC‐induced muscle atrophy, the exact mechanism by which the GC–GR system participates to the functional coupling between metabolic regulation and fibre size adjustment in skeletal muscle remains unsolved. A crosstalk between GR and mammalian target of rapamycin (mTOR) has been proposed as a mechanism for the fine‐tuning of the muscle volume.^7^ Of note, many pathological conditions characterized by muscle atrophy, for example, cachexia, sepsis, starvation, metabolic acidosis, and severe insulinopenia, are associated with an increase in circulating GC levels. Adrenalectomy or treatment with the GR antagonist RU486 attenuates muscle atrophy in various metabolic conditions and in GC‐induced myopathy.^8^, ^9^ Furthermore, GCs were shown to be required to stimulate muscle atrophy in acute diabetic rodents.^10^ Therefore, understanding the GC‐mediated regulation of metabolism–volume coupling in skeletal muscle is important for the treatment of not only muscle wasting but also these metabolic disorders.

In the last decade, the obestatin/GPR39 system has gained relevance as an autocrine pathway in the myogenic program.^11^, ^12^ Obestatin, a 23‐amino acid peptide derived from a polypeptide called preproghrelin, exerts an autocrine function through the G‐protein‐coupled receptor GPR39 to control the myogenic differentiation program.^11^ Obestatin is expressed in healthy skeletal muscle, and this expression is strikingly increased upon muscle injury.^11^, ^12^ Obestatin is co‐ordinately up‐regulated during the early stages of myogenesis, and its level remains sustained throughout terminal differentiation. The obestatin/GPR39 system up‐regulates the satellite cells marker Pax7, myogenic factors, and embryonic myosin heavy chain, thus participating to the regulation of the different stages of myogenesis: proliferation, migration, fusion, and myofibre growth.^12^ More generally, it enhances the regeneration process and the formation of larger‐calibre fibres in mouse skeletal muscle.^12^ In a human context, the obestatin/GPR39 system exerts an autocrine effect on the skeletal myogenic process promoting myogenic differentiation and fusion of human myoblasts.^13^ The molecular mechanisms underlying the action of obestatin involve both G‐protein‐dependent and G‐protein‐independent mechanisms linking the activated GPR39 receptor with distinct sets of effector proteins, thereby controlling the specificity and efficiency of the signals. The obestatin‐associated mitogenic action is determined by G‐protein‐dependent activation leading to intricate pathways related to the ERK1/2 and JunD axis. The transactivation of epidermal growth factor receptor (EGFR) through the β‐arrestin signal complex determines the cell cycle exit and the triggering and progression of obestatin‐dependent differentiation through a kinase hierarchy determined by the Akt, CAMKII, c‐Jun, and p38 axes.^13^ β‐Arrestins arbitrate cell fate between G‐protein‐dependent and β‐arrestin‐dependent signalling pathways to regulate the progression of the myogenic program. Added to its role in myogenesis and muscle regeneration, obestatin participates in the specification of muscle fibre identity by inducing skeletal muscle remodelling towards an oxidative phenotype and enhancing muscle strength. Obestatin acts through both class II histone deacetylases (HDAC)/myocyte enhancer factor‐2 (Mef2) and peroxisome proliferator‐activated receptor‐gamma coactivator 1α (PGC1α), thereby controlling the establishment of oxidative muscle fibres.^14^ These data position obestatin as a potential therapeutic candidate not only for muscle injury but also for skeletal muscle myopathies involving muscle degeneration/regeneration. Recent studies have demonstrated that obestatin can restore to some extent muscle integrity and function in a Duchenne muscular dystrophy (DMD) murine model: obestatin‐treated *mdx* mice display skeletal muscle remodelling towards an oxidative phenotype, with improved muscle strength and reduced skeletal muscle pathology.^15^ The potential of obestatin as a therapeutic approach is not only limited to an ameliorative treatment to slow the muscle damage but also as part of combinatorial treatment strategies. In cell transplantationtherapy, for example, myoblast‐based therapy by xenotransplanting primary human myoblasts into immunodeficient mice, obestatin not only enhances the efficiency of engraftment but also facilitates an even distribution of myoblasts within the host muscle, probably by enhancing migration.^14^ Of note, the obestatin/GPR39 system is able to counteract deregulations in proteostasis, for example, those associated with GC‐induced myotube atrophy, and to restore efficient basal homoeostasis.^16^ We provided evidence *in vitro* that obestatin triggers an antiatrophic signalling pathway, thereby protecting from experimentally induced atrophy, by regulating Akt, PKD/PKCμ, CAMKII, and AMPK signalling and its downstream targets in the control of protein synthesis, ubiquitin–proteasome system, and autophagy–lysosome system in mouse and human cells.^16^ These findings emphasize the function of the obestatin/GPR39 system in co‐ordinating a variety of pathways involved in the regulation of protein degradation during catabolic conditions.

In the present study, we have explored the role of the obestatin/GPR39 system in the GC‐induced muscle atrophy in both *in vivo* in mice and *in vitro* in human models. We identified FoxO transcription factors as key targets of the obestatin/GPR39 system to counteract the catabolic processes provoked by GCs. The activity of FoxOs is regulated at both transcriptional and post‐translational levels. At transcriptional levels, the expression of FoxO transcription factors is regulated by obestatin signalling via up‐regulation of the E3 ligase NEDD4 that determines the ubiquitin‐dependent degradation of Krüppel‐like factor 15 (KLF15). On the other hand, specific patterns of post‐translational modifications of FoxO transcription factors, including FoxO4 phosphorylation, play a key role in directing FoxO activity in response to obestatin. Both processes determine protein degradation and autophagy via the regulation of, for example, MAFbx, MuRF1, LC3, p62, and cathepsin L. Simultaneously, obestatin activates Akt/mTOR signalling and positively modulates myofibre size, leading to a substantial restoration of muscle phenotype in GC‐treated mice. We propose that the co‐ordinated crosstalk between GR and obestatin signalling can regulate the fine‐tuning of muscle mass and thus could represent a potential therapeutic target for muscle wasting.

## Materials and methods

### Materials

Rat/mouse obestatin and human obestatin were obtained from BCN Peptides (Barcelona, ES). The rat/mouse haemagglutinin (HA)‐tagged obestatin (HA‐obestatin) was obtained from Biomedal (Sevilla, ES). Antibodies used are listed in Supporting Information, *Table* *S1*. All other chemical reagents were from Sigma Chemical Co. (St. Louis, MO, USA).

### Animals

This study used 6‐week‐old female Swiss mice (25 g) obtained from the breeding animal facilities of the University of Santiago de Compostela (Santiago de Compostela, ES). Mice were housed in 12 h light/12 h dark cycles with free access to standard mice chow diet and water. Experiments were performed in accordance with the University of Santiago de Compostela guidelines for animal handling and animal care as determined by the University of Santiago de Compostela Animal Care Committee according to the guidelines of the Spanish Royal Decree 53/2013, Directive 2010/63/EU, and FELASA guidelines.

### Dexamethasone and obestatin dosing

For systemic obestatin administration and dispersal study, mice were injected intraperitoneally with saline [vehicle control, 0.9% NaCL (w/v), 100 μL] or HA‐obestatin solution in saline (500 nmol/kg body weight, 100 μL) inserting the needle of a 29‐gauge syringe (BD Micro‐Fine insulin syringe) into the peritoneal cavity. Mice were euthanized 2 h after treatment, and tibialis anterior (TA), gastrocnemius, soleus, and diaphragm muscles were excised and processed for subsequent immunofluorescence analyses. For studies in the dexamethasone (Dexa; dexamethasone‐water soluble)‐induced skeletal muscle atrophy model, mice were assigned to one of the following experimental groups (*n* = 8 per group): (i) control group, (ii) Dexa‐treated group, (iii) Dexa + vehicle‐treated group, and (iv) Dexa + obestatin‐treated group. The control group was not exposed to any experimental intervention, thus serving as a neutral comparison for study groups. The Dexa‐treated group was injected intraperitoneally with Dexa, dissolved in water (15 mg/kg body weight, 100 μL), every 24 h during 10 days. The Dexa + vehicle group was injected intraperitoneally with Dexa under the same conditions (15 mg/kg body weight/24 h, 10 days) and, as from the fourth day, received intraperitoneal injections of saline [vehicle control, 0.9% NaCL (w/v), 100 μL] every 72 h for 7 days. The obestatin‐treated group was injected with Dexa (100 μL, 15 mg/kg body weight each 24 h for 10 days), and, as from the fourth day, intraperitoneal injections of obestatin solution in saline (500 nmol/kg body weight, 100 μL) were co‐administered every 72 h for 7 days. Mice were euthanized 10 days after the Dexa treatment, and skeletal muscle samples [TA, extensor digitorum longus (EDL), gastrocnemius, soleus, and diaphragm muscles] were excised and processed for subsequent analyses. A schematic representation of this protocol is presented in *Figure* 2A.

### Cell culture and differentiation

Human myogenic cell line, KM155C25 Clone 48 (KM155C25 cells), was obtained from the platform for immortalization of human myoblasts of the Center of Research in Myology in Paris (France), who developed it from a biopsy obtained through MYOBANK, a partner in the EU network EuroBioBank (gracilis muscle, donor age 25 years), following the protocols previously described.^16^, ^17^ Immortalized human myoblasts maintain their capacity to differentiate both *in vitro* and *in vivo* after transplantation into the regenerating muscles of immunodeficient mice.^17^, ^18^ KM155C25 cells were cultivated in GM‐containing Medium 199:DMEM (1:5, v/v; Lonza, Pontevedra, ES) supplemented with 20% foetal bovine serum (v/v), 25 μg/μL fetuin, 5 ng/mL human epidermal growth factor, 0.5 ng/mL basic fibroblast growth factor, and 50 μg/mL gentamicin (Invitrogen; CA, USA) as described previously (Cid‐Díaz T *et al*. 2017). Differentiation into myotubes was initiated at 90% confluence by switching to DM [Medium 199:DMEM (1:5, v/v) supplemented with 50 μg/mL gentamicin] for 7 days unless otherwise stated. Human myotubes were treated with Dexa (1 μM) for 24 h in the presence or absence of obestatin (10 nM) or with insulin (100 nM) as a positive control of atrophy protection.

### Histological analysis

Muscle samples were mounted in tissue freezing medium (tragacanth gum) and snap frozen in nitrogen‐cooled isopentane. The sections, 5 μm thick, were mounted on HistoBond adhesion microslides (Marienfeld, Lauda‐Königshofen, DE). Skeletal muscle sections were stained with haematoxylin and eosin (HE) to quantify myofibre area using NIH Image Software ImageJ64 (National Institutes of Health, Bethesda, MD, USA). For immunofluorescence analysis, skeletal muscle sections were permeabilized and blocked with PBST [1% Triton X‐100, 1% Tween 20, 5% heat‐inactivated goat serum, 0.2% bovine serum albumin in phosphate‐buffered saline (PBS)] for 30 min and incubated with primary antibodies diluted in PBST overnight at 4°C, washed with PBS, and then incubated with a mixture of appropriate secondary antibodies for 45 min at 37°C. 4′,6‐Diamidino‐2‐phenylindole (DAPI) was used to counterstain the nuclei (Invitrogen). The digital images of the cell cultures were acquired with a Leica TCS SP8 spectral confocal microscope (Leica Microsystems, Heidelberg, DE).

### Small interfering RNA silencing of gene expression

To ablate KLF15 or Nedd4 expression in human KM155C25 myotubes, small interfering RNA (siRNA) specifically targeting human KLF15 (sc‐45567, Santa Cruz Biotechnology, CA, USA) or Nedd4‐1 (sc‐41079, Santa Cruz Biotechnology) was used. siRNA duplexes targeting FoxO4 were selected from ON‐TARGETplus SMARTpool siRNA from Thermo Fisher Scientific (Dharmacon; human FoxO4: AGUCAUGCCUGGAAGCUUU, GAGAAGCGACUGACACUUG, GGAAAUACCAGCUUCAGUC, CAACGAGGCCACCGGCAAA). An ON‐TARGETplus nontargeting siRNA was used as a control for all siRNA experiments. Human KM155C25 myotubes were transfected at Day 6 post‐differentiation with Lipofectamine 2000 (Invitrogen), following the manufacturer's instructions.

### SDS‐PAGE and western blot analysis

Cell or tissue samples were directly lysed in ice‐cold RIPA buffer [50 mM Tris‐HCl (pH 7.2), 150 mM NaCl, 1 mM EDTA, 1% (v/v) NP‐40, 0.25% (w/v) Na‐deoxycholate, protease inhibitor cocktail, phosphatase inhibitor cocktail]. The lysates were clarified by centrifugation (14 000× *g* for 15 min at 4°C), and the protein concentration was quantified using the QuantiProTM BCA assay kit. For immunoblotting, equal amounts of protein were fractionated by SDS‐PAGE and transferred onto nitrocellulose membranes. Immunoreactive bands were detected by enhanced chemiluminescence (Pierce ECL Western Blotting Substrate; Thermo Fisher Scientific, Pierce, Rockford, IL, USA).

### Co‐immunoprecipitation assays

Following treatment [DM, DM + Dexa (1 μM, 24 h), DM + Dexa (1 μM, 24 h) + obestatin (10 nM, 24 h), DM + obestatin (10 nM, 24 h) in the absence or presence of 15 μM MG132 for 6 h], KM155C25 myotube cells were washed twice with ice‐cold PBS and lysed in co‐immunoprecipitation lysis buffer (50 mM Tris, 100 mM NaCl, 5 mM EDTA, 50 mM NaF, 1% Triton X‐100, 10 mM glycerol phosphate, 200 μM sodium orthovanadate, 2.5 mM sodium pyrophosphate plus protease inhibitors). KLF15 was immunoprecipitated using mouse monoclonal anti‐KLF15 antibody coupled to protein A Dynabeads according to the instructions provided by the manufacturer (Thermo Fisher Scientific, Life Technologies, Rockford, IL, USA). The washed immunoprecipitates were subjected to western blot analysis using the indicated antibodies.

### Statistics

All values are presented as mean ± standard error of the mean (SEM). A Shapiro–Wilks normality test was performed for each data set. *t*‐tests were carried out for comparisons between two samples. Unpaired *t*‐test was used to assess the statistical significance of one‐way or two‐way analysis when the test statistic followed a normal distribution. *P* < 0.05 was considered as statistically significant (*^,&,#^ *P* < 0.05).

### Study approval

All animal experiments were approved by the animal experimentation committee of the University of Santiago de Compostela (Santiago de Compostela, Spain; No. 15010/17/005).

## Results

**Figure 1:**
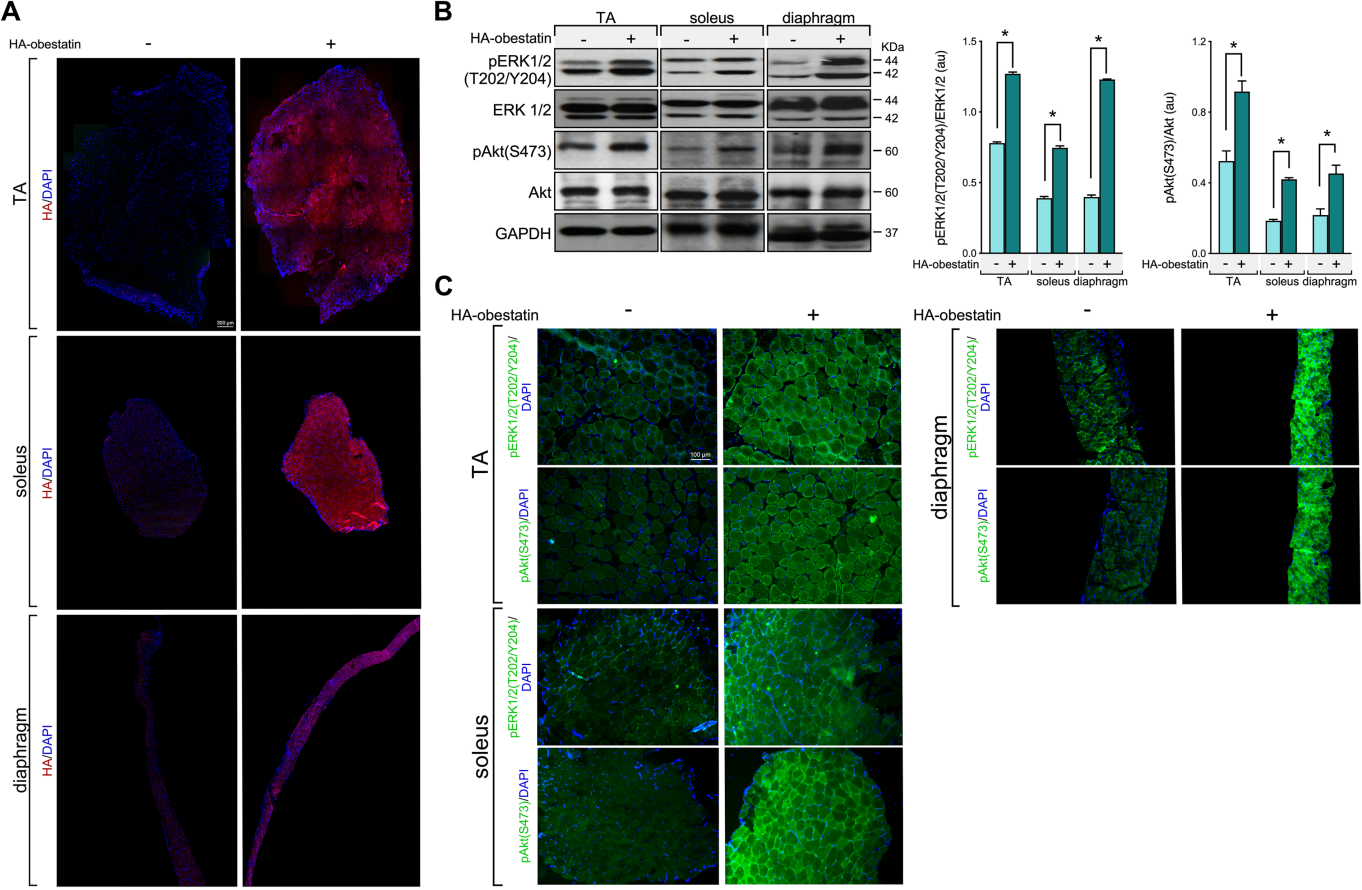
Analysis of delivery of obestatin peptide administration via intraperitoneal injection to body musculature. Haemagglutinin (HA)‐tagged obestatin (500 nmol/kg body weight each; *n* = 5) or vehicle (control; *n* = 5) was administrated to mice via intraperitoneal for 2 h as described in the Materials and methods section. (*A*) Representative immunostaining for HA in transverse cryosections of TA, soleus, and diaphragm muscles after 2 h of treatment with HA‐tagged obestatin or vehicle. (*B*) Analysis of ERK1/2 and Akt activity in TA, soleus, and diaphragm muscles 2 h after HA‐tagged obestatin administration. Immunoblots are representative of the mean value (*n* = 5 per group). ERK1/2 and Akt activities are in arbitrary units (au). Data are expressed as mean ± SEM obtained from intensity scans (*P < 0.05). (*C*) Representative immunostaining for pERK1/2 and pAkt in transverse cryosections of TA, soleus, and diaphragm muscles after 2 h of treatment with HA‐tagged obestatin or vehicle.

**Figure 2:**
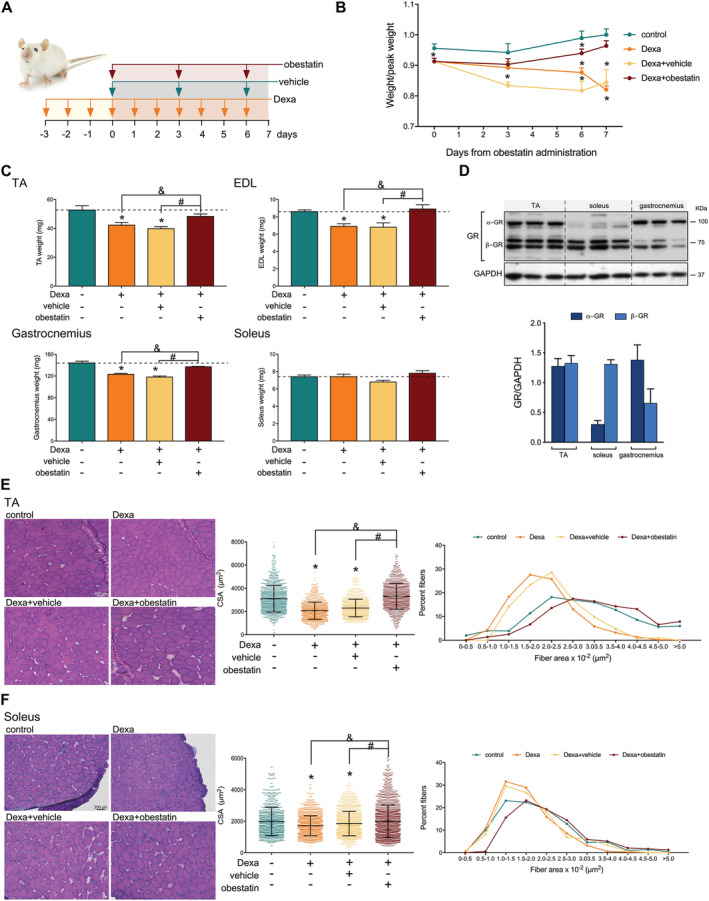
Obestatin treatment restores muscle phenotype in dexamethasone‐induced skeletal muscle atrophy model. (*A*) Schematic diagram of the intraperitoneal administration of dexamethasone (Dexa; 15 mg/kg body weight every 24 h, 10 days; *n* = 8), obestatin (500 nmol/kg body weight every 72 h, 7 days; *n* = 8), or vehicle (PBS every 72 h, 7 days; *n* = 8) in Swiss mice. (*B*) Time course of body weight of control, Dexa‐treated, Dexa + vehicle‐treated, and Dexa + obestatin‐treated mice. (*C*) TA, EDL, gastrocnemius, and soleus muscle weight in control, Dexa‐treated, Dexa + vehicle‐treated, and Dexa + obestatin‐treated mice. (*D*) Immunoblot analysis of GR levels in mouse TA, soleus, and gastrocnemius muscle. GR data were expressed relative to GAPDH as arbitrary units (mean ± SEM) obtained from intensity scans. (*E*) *Left panel*, representative haematoxylin and eosin staining of cryosections of TA muscle from control, Dexa‐treated, Dexa + vehicle‐treated, and Dexa + obestatin‐treated mice. *Middle panel*, cross‐sectional area (CSA) of muscle fibres from TA muscles obtained from control, Dexa‐treated, Dexa + vehicle‐treated, and Dexa + obestatin‐treated mice. *Right panel*, distribution of fibre diameter from TA muscles obtained from control, Dexa‐treated, Dexa + vehicle‐treated, and Dexa + obestatin‐treated mice. Data are expressed as % of myofibres per class of fibre diameter. (*F*) *Left panel*, representative haematoxylin and eosin staining of cryosections of soleus muscle from control, Dexa‐treated, Dexa + vehicle‐treated, and Dexa + obestatin‐treated mice. *Middle panel*, cross‐sectional area (CSA) of muscle fibres from TA muscles from control, Dexa‐treated, Dexa + vehicle‐treated, and Dexa + obestatin‐treated mice. *Right panel*, distribution of fibre diameter from TA muscles from control, Dexa‐treated, Dexa + vehicle‐treated, and Dexa + obestatin‐treated mice. Data are expressed as % of myofibres in each class of fibre diameter. In (*E*) and (*F*), data are expressed as mean ± SEM (*n* = 8 animals per group). *^,&,#^ *P* < 0.05.

**Figure 3:**
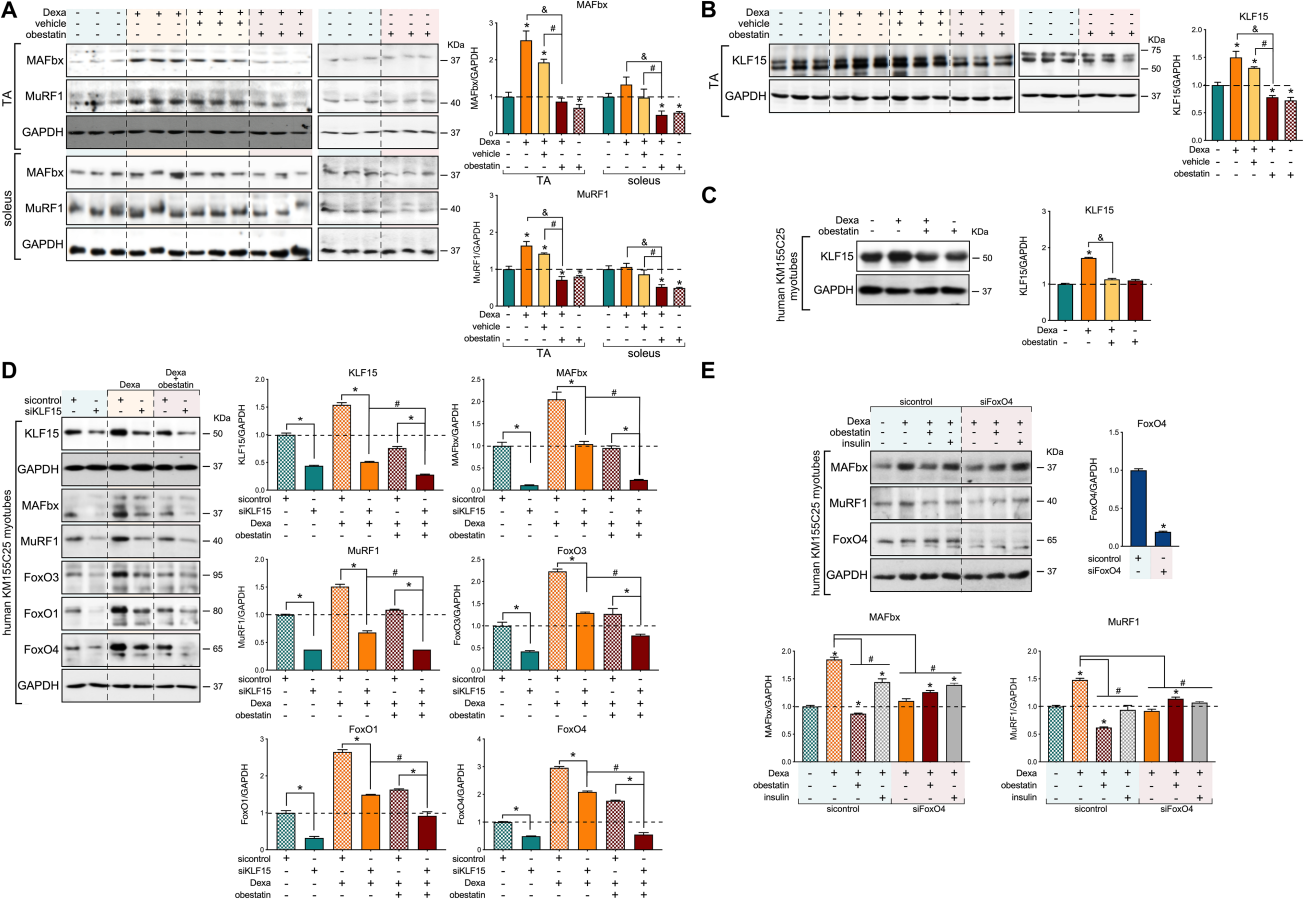
Regulation of atrogenes and FoxOs by KLF15 in dexamethasone‐induced skeletal muscle atrophy. (*A*) Immunoblot analysis of MAFbx and Murf1 levels in mouse TA and soleus muscle after intraperitoneal administration of Dexa (15 mg/kg body weight every 24 h, 10 days; *n* = 8), obestatin (500 nmol/kg body weight every 72 h, 7 days; *n* = 8), or vehicle (PBS every 72 h, 7 days; *n* = 8) in Swiss mice. (*B*) Immunoblot analysis of KLF15 levels in mouse TA muscle after intraperitoneal administration of Dexa (15 mg/kg body weight every 24 h, 10 days; *n* = 8), obestatin (500 nmol/kg body weight every 72 h, 7 days; *n* = 8), or vehicle (PBS every 72 h, 7 days; *n* = 8) in Swiss mice. (*C*) Immunoblot analysis of KLF15 in human KM155C25 myotubes under DM, DM + Dexa (1 μM, 24 h), DM + Dexa (1 μM, 24 h) + obestatin (10 nM, 24 h), or DM + Dexa (1 μM, 24 h) + insulin (100 nM, 24 h) (*n* = 4). (*D*) After transfection with specific KLF15 siRNA, KM155C25 myotubes were treated with Dexa (1 μM) or Dexa (1 μM) + obestatin (10 nM) for 24 h. The effect of KLF15 siRNA was evaluated on MAFbx, Murf1, FoxO3, FoxO1, and FoxO4 expression (*n* = 4). (*E*) Immunoblot analysis of MAFbx, Murf1, and FoxO4 in human KM155C25 myotubes transfected with specific FoxO4 siRNA under DM, DM + Dexa (1 μM, 24 h), or DM + Dexa (1 μM, 24 h) + obestatin (10 nM, 24 h). In (*A*) and (*B*), protein level is expressed as fold of control. Data are expressed as mean ± SEM obtained from intensity scans. *^,#,&^ *P* < 0.05. In (*C*)–(*E*), immunoblots are representative of the mean value (*n* = 4). Data were expressed as mean ± SEM obtained from intensity scans. Data are expressed as mean ± SEM. *^,#,&^ *P* < 0.05.

To examine the effect of obestatin signalling on GC‐induced muscle atrophy, we first tested the adequate delivery of obestatin peptide to body musculature. Intraperitoneal injection of HA‐tagged obestatin in mice resulted in marked increase of HA‐tag in TA, soleus, and diaphragm muscles (*Figure* 1A). Consistent with this, HA‐tagged obestatin treatment resulted in elevated phosphorylation levels of ERK1/2 [pERK1/2(T202/Y204)] and Akt [pAkt(S473)] 2 h after the onset of peptide administration in TA, soleus, and diaphragm muscles (*Figure* 1B and 1C). We then utilized a mouse model of GC‐induced muscle wasting by exogenous administration of high dose of Dexa to determine the interaction of GR and obestatin signalling in the regulation of metabolism–volume coupling in skeletal muscle. In particular, skeletal muscle decline was promoted prior to obestatin administration by treatment with Dexa (15 mg/kg body weight, each 24 h) for 3 days prior to intraperitoneal injection of obestatin (500 nmol/kg body weight, each 72 h) for 7 days (*Figure* 2A). Indeed, the Dexa‐treated mice displayed a significant decrease of relative body weight compared with control mice 3 days after Dexa administration (designated as Day 0; *Figure* 2B). The divergence in the Dexa‐treated population occurred about 6 days following obestatin treatment, with the Dexa‐treated or Dexa + vehicle‐treated groups showing a significant loss of total body weight, compared with the control group. No loss of weight was observed, however, in the Dexa + obestatin‐treated group (*Figure* 2B). The increase of total body weight included an increase in skeletal muscle mass, as demonstrated by significant increase in type II‐rich TA, EDL, and gastrocnemius muscle mass in the Dexa + obestatin‐treated mice compared with the Dexa‐treated or Dexa + vehicle‐treated groups (*Figure* 2C). In clear contrast, the mass of the type I‐rich soleus muscle was unchanged (*Figure* 2C). Cross‐sectional area (CSA) analysis showed a shift towards smaller myofibre CSA in the TA muscle and, to a lesser extent, in the soleus muscle in both the Dexa‐treated and Dexa + vehicle‐treated groups. The Dexa + obestatin group revealed a significant recovery of muscle when compared with Dexa groups in TA muscle and, to a minor extent, in the soleus muscle (*Figure* 2E and 2F, respectively). CSA distribution of myofibres supported this fact; the leftward shift in myofibre size was observed in the Dexa‐treated and Dexa + vehicle‐treated groups, but not in the Dexa + obestatin group showing values closely related to the control mice in TA muscles. In contrast, the difference in the size was far less pronounced in the soleus fibres, highlighting the effect of obestatin treatment in myofibre size. The mechanism of this specificity can be related to the relatively low α‐isoform of the GR (α‐GR) levels observed in the type I‐rich soleus muscle compared with the type II‐rich TA muscles (*Figure* 2D).^19^, ^20^, ^21^ Indeed, Dexa induction of the E3 ubiquitin ligases MAFbx and MuRF1 was observed in α‐GR‐rich TA muscles with reduced or no impact on soleus fibres (*Figure* 3A). When obestatin was administrated, the levels of MAFbx and MuRF1 were efficiently down‐regulated in TA muscles (*Figure* 3A). In this model, obestatin signalling also suppressed the Dexa‐induced expression of KLF15 in TA muscle, a transcription factor induced by various stressors, including GCs, to regulate muscle catabolism via the regulation of the E3 ubiquitin ligases MAFbx and MuRF1 (*Figure* 3B).^7^, ^22^, ^23^, ^24^ Similarly, we identified the Dexa‐mediated up‐regulation of KLF15 protein expression and confirmed its down‐regulation by obestatin signalling in human KM155C25 myotubes (*Figure* 3C). We then asked whether the transcription network activated by GRs was dependent on KLF15. For that purpose, we tested the effect of the down‐regulation of KLF15 by specific siRNA (siKLF15) on protein expression of MAFbx, MuRF1, FoxO3, FoxO1, and FoxO4 (*Figure* 3D). In human KM155C25 myotubes, KLF15 knockdown diminished the Dexa‐dependent protein induction of all these GR target proteins (*Figure* 3D). Note that Dexa + obestatin vs. Dexa resulted in additive effect on siKLF15 (*Figure* 3D). MAFbx and MuRF1 were originally identified as FoxO target genes,^25^, ^26^ and KLF15 was shown to regulate FoxO expression.^7^ Indeed, the siRNA against FoxO4, the main obestatin‐responsive FoxO isoform,^16^ significantly decreased the MuRF1 and MAFbx expression in Dexa‐stimulated cells (*Figure* 3E). Moreover, siFoxO4 experiments partially restored the MuRF1 and MAFbx expression in Dexa + obestatin‐treated cells (*Figure* 3E). These results strongly indicate the critical involvement of obestatin signalling on the GR‐KLF15 cascade to regulate the Dexa‐mediated up‐regulation of E3 ubiquitin ligase expression.

**Figure 4:**
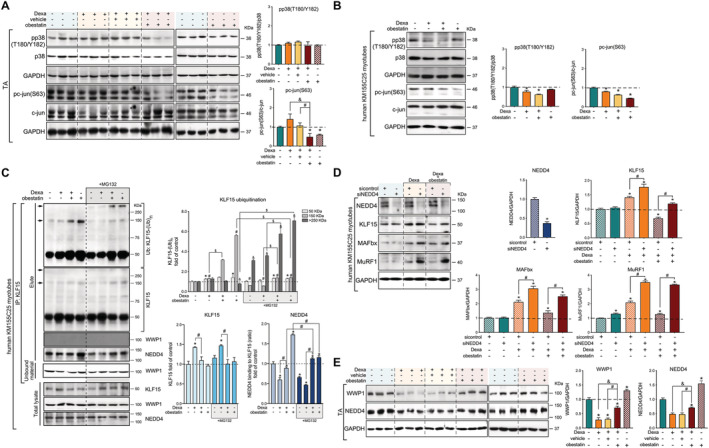
Obestatin signalling regulates the KLF15‐triggered expression program via the E3 ubiquitin ligase NEDD4. (*A*) Immunoblot analysis of pp38(T180/Y182) and pc‐jun(S63) in TA muscle after intraperitoneal administration of Dexa (15 mg/kg body weight each 24 h, 10 days; *n* = 8), obestatin (500 nmol/kg body weight each 72 h, 7 days; *n* = 8), or vehicle (PBS each 72 h, 7 days; *n* = 8) in Swiss mice. Protein level is expressed as fold of control. Data are expressed as mean ± SEM obtained from intensity scans (*^,#,&^ *P* < 0.05). (*B*) Immunoblot analysis of pp38(T180/Y182) and pc‐jun(S63) in human KM155C25 myotubes under DM, DM + Dexa (1 μM, 24 h), or DM + Dexa (1 μM, 24 h) + obestatin (10 nM, 24 h). Immunoblots are representative of the mean value (*n* = 4). Data were expressed as mean ± SEM obtained from intensity scans. (*P < 0.05). (*C*) Co‐immunoprecipitation assays used to determine the KLF15‐interacting proteins in human KM155C25 myotubes under DM, DM + Dexa (1 μM, 24 h), DM + Dexa (1 μM, 24 h) + obestatin (10 nM, 24 h), or DM + obestatin (10 nM, 24 h) stimulation in the absence or presence of MG132 (15 μM). The KLF15 complex was co‐immunoprecipitated with anti‐KLF15 affinity beads and analysed by western blot using anti‐ubiquitin, anti‐KLF15, anti‐WWP1, and anti‐NEDD4 antibodies. Arrows indicate the main polyubiquitinated forms of KLKF15, for example, 150 and 250 kDa. Immunoblots are representative of the mean value (*n* = 4). Data are expressed as mean ± SEM obtained from intensity scans (*^,#,&,$^ *P* < 0.05). (*D*) After transfection with specific NEDD4 or control siRNA, KM155C25 myotubes were treated with Dexa (1 μM) or Dexa (1 μM) + obestatin (10 nM) for 24 h. The effect of NEDD4 siRNA was evaluated on KLF15, MAFbx, and Murf1 (*n* = 4). Data are expressed as mean ± SEM obtained from intensity scans (*^,#^ *P* < 0.05). (*E*) Immunoblot analysis of the WWP1 and NEDD4 protein levels in TA muscle after intraperitoneal administration of dexamethasone (Dexa; 15 mg/kg body weight each 24 h, 10 days; *n* = 8), obestatin (500 nmol/kg body weight each 72 h, 7 days; *n* = 8), or vehicle (PBS each 72 h, 7 days; *n* = 8) in Swiss mice. Protein level is expressed as fold of control. Immunoblots are representative of the mean value. Data are expressed as mean ± SEM obtained from intensity scans (*^,&,#^ *P* < 0.05).

An important question is how KLF15 can be shut down by obestatin signalling in skeletal muscle. p38 MAPK^27^ and c‐Jun^28^ were identified as negative modulators of the GR‐mediated KLF15 transcription. However, obestatin suppressed Dexa‐mediated phosphorylation of c‐Jun at S63 in TA muscles, without a significant alteration in p38 phosphorylation at T180/Y182 (*Figure* 4A). These effects were confirmed in human KM155C25 myotubes (*Figure* 4B). Post‐translational modification of KLF15 protein by the E3 ubiquitin ligases is one of the key regulatory steps that guides protein degradation through regulation of proteasome activity. WW domain‐containing E3 ubiquitin protein ligase 1 (WWP1) and neural precursor cell‐expressed developmentally down‐regulated 4 (NEDD4) were previously shown to interact with other members of the KLF family.^29^ Co‐immunoprecipitation analysis revealed that NEDD4, but not WWP1, interacted with KLF15 in human KM155C25 myotubes (*Figure* 4C). Dexa increased the protein expression but failed to modify the polyubiquitination of KLF15 [KLF15‐(Ub)_n_]. In clear contrast, obestatin inhibited Dexa‐mediated KLF15 expression by promoting the polyubiquitination of KLF15‐(Ub)_n_ (mw ≅ 150 kDa; *Figure* 4C). Moreover, treatment of the cells with the proteasome inhibitor MG132 enhanced the amount of KLF15 ubiquitin conjugates in both Dexa + obestatin‐stimulated and obestatin‐stimulated human myotubes [KLF15‐(Ub)_n_ mw > 250 kDa; *Figure* 4C]. Such an enhancement was paralleled by NEDD4 recruitment onto KLF15 (*Figure* 4C). Thus, obestatin negatively modulates KLF15 function, most possibly by altering the protein ubiquitination profile, the linkage type, and length of ubiquitin chains through NEDD4 and linking the substrates to downstream processes.

We next examined the effect of knocking down the expression of NEDD4 by siRNA (siNEDD4) on KLF15 activity from human KM155C25 myotubes. NEDD4 siRNA‐mediated knockdown increased the response to Dexa of a number of GC‐inducible proteins, including KLF15, MAFbx, and MuRF1, when compared with control siRNA (*Figure* 4D). Treatment with obestatin clearly inhibited the induction by DEXA of KLF15, MAFbx, and MuRF1 proteins in human myotubes transfected with control siRNA, and such a process was significantly reversed by siNEDD4 (*Figure* 4D). In TA muscle, protein expression of NEDD4 was suppressed by Dexa, and obestatin suppressed at least partly, but significantly, this Dexa‐mediated down‐regulation of NEDD4 (*Figure* 4E). This positive modulation by obestatin was also observed on WWP1 protein expression profile (*Figure* 4E). Taken together, these data indicate that obestatin regulates the liaison molecule for GR in the induction of atrogenes, KLF15, through the control of NEDD4.

**Figure 5:**
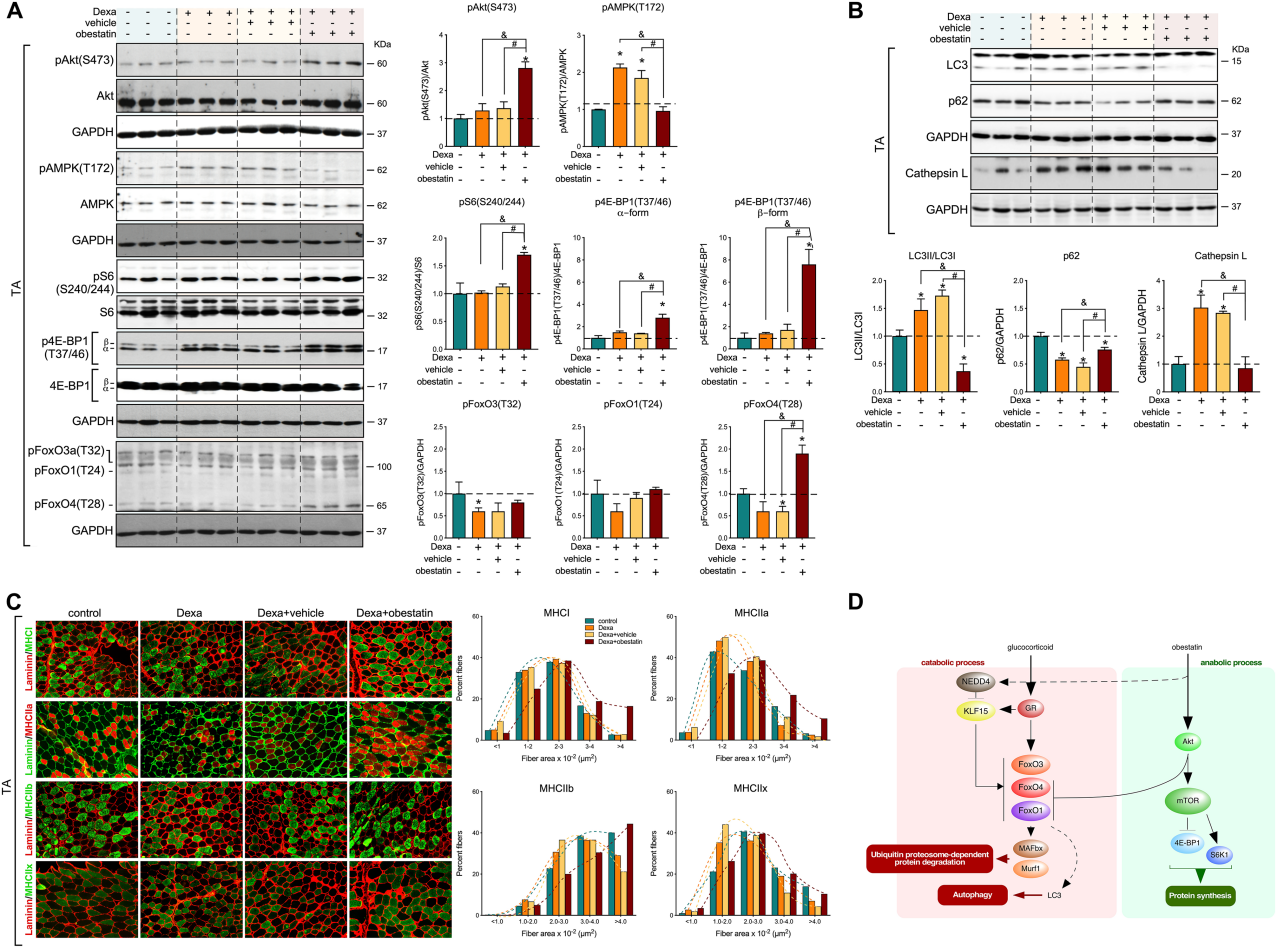
Obestatin treatment protects from atrophy with induction of protein synthesis signalling. (*A*) Immunoblot analysis of pAkt(S473), Akt, pAMPK(T172), AMPK, pS6(S240/244), S6, p4E‐BP1(T37/46), 4E‐BP1, pFoxO3a(T32), pFoxO1(T24), and pFoxO4(T28) in TA muscle after intraperitoneal administration of Dexa (15 mg/kg body weight each 24 h, 10 days; *n* = 8), obestatin (500 nmol/kg body weight every 72 h, 7 days; *n* = 8), or vehicle (PBS every 72 h, 7 days; *n* = 8) in Swiss mice. Protein level is expressed as fold of control. Data are expressed as mean ± SEM obtained from intensity scans (*^,&,#^ *P* < 0.05). (*B*) Immunoblot analysis of the LC3, p62, and cathepsin L in TA muscle after intraperitoneal administration of Dexa (15 mg/kg body weight every 24 h, 10 days; *n* = 8), obestatin (500 nmol/kg body weight every 72 h, 7 days; *n* = 8), or vehicle (PBS every 72 h, 7 days; *n* = 8) in Swiss mice. Protein level is expressed as fold of control. Data are expressed as mean ± SEM obtained from intensity scans (*^,&,#^ *P* < 0.05). (*C*) *Left panel*, representative images of control‐treated, Dexa‐treated, Dexa + vehicle‐treated, and Dexa + obestatin‐treated TA muscles showing MHC expression. Serial muscle cross sections were incubated with a primary antibody against MHCI, MHCIIa, MHCIIb, or MHCIIx. *Right panel*, frequency distribution histograms of cross‐sectional areas (CSA; μm^2^). Data are shown as mean ± SEM (*n* = 8 per group). (*D*) Schematic model of the crosstalk between the GC‐associated catabolic processes and the obestatin‐triggered anabolic processes in skeletal muscle.

We then examined the effects of Dexa and obestatin on protein levels and phosphorylation status of AMPK and Akt, the upstream activator of mTOR, and mTOR downstream effectors S6 and 4E‐BP1, in the mouse GC‐induced atrophy model. In TA muscle, treatment with Dexa increased the phosphorylation of AMPK [pAMPK(T172)], without significant alteration in the phosphorylation of Akt [pAkt(S473)], FoxO family [pFoxO3(T32), pFoxO1(T24), pFoxO4(T28)], S6 [pS6(S240/244)], and 4E‐BP1 [p4E‐BP1(T37/41)] (*Figure* 5A). In clear contrast, the levels of pAkt(S473), pFoxO4(T24), pS6(S240/244), and p4E‐BP1(T37/41) were significantly increased when obestatin was administrated, while pAMPK(T172) was inhibited (*Figure* 5A). In this model, Dexa administration increased the expression of lysosomal enzyme cathepsin L and the conversion rate of the microtubule‐associated protein 1 light chain 3 isoform I (LC3I) to LC3 isoform II (LC3II), with significant decline in the amount of p62 protein indicating autophagic degradation activity (*Figure* 5B). In clear contrast, treatment with obestatin reversed the effects of Dexa (*Figure* 5B). Significant accumulation of p62 protein was seen compared with Dexa treatment alone indicating a block in autophagy (*Figure* 5B). Furthermore, obestatin treatment was associated with a reduction in the expression of LC3II and cathepsin L, further supporting an inactivation of autophagy (*Figure* 5B). Histological examination of the TA muscle demonstrated type II fibre‐dominant atrophy in the Dexa and Dexa + vehicle groups. This atrophic effect was also characterized by fast‐twitch type IIb/IIx glycolytic muscle fibre loss and reduced impact on slow‐twitch type I and fast‐twitch type IIa oxidative muscle fibres (*Figure* 5C). In contrast, the Dexa + obestatin group showed less impairment in the TA muscle by preventing of atrophy and type II fibre loss (*Figure* 5C). CSA analysis of myofibres supported this observation, because a leftward shift in the fast‐twitch type IIb/IIx glycolytic myofibre size was observed in the Dexa and Dexa + vehicle groups, but not in the Dexa + obestatin group (*Figure* 5C). Dexa‐treated muscles showed limited or no impact on fast‐twitch type IIa oxidative and slow‐twitch type I oxidative fibres, respectively (*Figure* 5C). Of note, there were significant differences in the size of slow‐twitch type I, fast‐twitch type IIa oxidative, and fast‐twitch IIb glycolytic muscle fibres in the obestatin‐treated group compared with the treatment with Dexa alone or Dexa + vehicle and even to the control group (*Figure* 5C). Therefore, we conclude that the interplay among Akt, mTOR, KLF15, and FoxO4 regulates two of the main proteolytic systems, the ubiquitin–proteasome and autophagy–lysosome systems, and the signalling associated with protein synthesis in response to the obestatin/GPR39 system to counteract the GR‐dependent catabolic processes.

## Discussion

In skeletal muscle, anabolic and catabolic pathways are finely tuned in a co‐ordinated manner to regulate myofibre size and total muscle mass. Pathological conditions associated with increased levels of circulating GC distort this balance, leading to a generalized decrease in myofibre size and to muscle atrophy. In this report, we show that the activation of the obestatin/GPR39 system protects from GC‐induced atrophy by regulation of Akt and KLF15 signalling and its downstream targets in the control of protein synthesis, ubiquitin–proteasome system, and autophagy–lysosome system. FoxO transcription factors are common elements that act as critical liaison molecules for protein degradation and autophagy. The pattern of post‐translational modifications of FoxO transcription factors, including specific FoxO4 phosphorylation, triggers FoxO activity in response to obestatin. At transcriptional levels, the expression of FoxO family is regulated by the ubiquitin‐dependent degradation of KLF15 via up‐regulation of the E3 ligase NEDD4. Moreover, obestatin activates Akt/mTOR signalling and modulates positively myofibre size, leading to a substantial restoration of muscle phenotype in GC‐treated atrophic mice. The co‐ordinated crosstalk between GR and the obestatin/GPR39 system represents a novel mechanism for the fine‐tuning of muscle mass and, in this way, a potential therapeutic target to counteract muscle wasting.

The GC‐induced muscle atrophy is a result of the functional cooperativity among GR, FoxOs, and KLF15 as critical liaison molecules aimed at increasing protein degradation.^1^, ^7^ A significant question raised in this study is how the GR‐driven proteolytic cascades can be shut down by obestatin signalling. In our model, obestatin signalling negatively modulates GR‐mediated gene expression, and this action derives from the regulation of FoxO transcription factors at both translational and post‐transcriptional levels. At post‐translation level, FoxO family is negatively regulated by the Akt pathway in response to obestatin. Phosphorylation of FoxO factors by Akt causes the sequestration of FoxO in the cytoplasm, thereby preventing FoxO factors from transactivating their target genes in the nucleus.^30^, ^31^, ^32^ Our results show that neither FoxO1 nor FoxO3 is involved in the response to obestatin via phosphorylation by Akt activation. In contrast, FoxO4 is the main obestatin‐responsive isoform, thus supporting an isoform‐specific mode of regulation previously described in human myotubes.^16^ At transcriptional level, the effect of obestatin on FoxO factors is mediated by a down‐regulation of KLF15 expression. It is worth to underline that the E3 ubiquitin ligase responsible for KLF15 polyubiquitination in response to obestatin is NEDD4. This fact is supported by the results of the immunoprecipitation assays that demonstrate that NEDD4 interacts specifically with KLF15 and that this interaction is increased in response to obestatin. Moreover, siRNA targeting NEDD4 suppresses the potential of obestatin to regulate KLF15 and the atrogenes during GC treatment. Interestingly, in skeletal muscle, NEDD4 has been reported to be involved in the generation of atrophy under very specific conditions in skeletal muscle such as in disuse (unloading or denervation)‐induced muscle atrophy.^33^, ^34^, ^35^, ^36^, ^37^, ^38^ However, the NEDD4 expression under obestatin signalling correlates with a regression of the atrophic process, concomitant with a decreased expression of KLF15 protein. Thus, we propose that NEDD4 controls the ubiquitination, or stability, or both, of KLF15. This protein complex determines the KLF15 activity, for example, control of FoxO and atrogenes expression. In contrast to changes in subcellular localization achieved by Akt activation, which are rapidly reversible, changes in FoxO protein levels are more permanent, which may have profound impacts on FoxO functions in the progress of muscle wasting. Within the GR‐triggered proteostasis network, the activation of obestatin signalling blocks the lysosome–autophagy system. In fact, the reduction of LC3 lipidation (LC3II) and the accumulation of p62 together with a reduced cathepsin L protein level limit the final degradation steps of up‐regulated autophagy. It is well established that FoxO proteins induce autophagy not only through transactivation of autophagic genes but also by interacting with autophagic proteins and by epigenetically regulating autophagic activity.^39^, ^40^, ^41^, ^42^ In this way, the post‐translational modification of FoxOs may down‐regulate autophagy. Thus, in skeletal muscle, the lysosomal and proteasomal pathways for protein degradation can be co‐ordinately regulated upon activation of obestatin signalling. The simultaneous inhibition of these two proteolytic pathways by obestatin signalling presumably plays the role of safeguards against the loss of different cell components, for example, myofibrillar proteins, and organelles, especially mitochondria. Both modes of regulating FoxO activity through obestatin signalling with the enhancement of the protein synthesis pathway, for example, the activation of S6K1 and the eIF4E availability, through phosphorylation of 4E‐BP1,^43^ restore basal homoeostasis conditions. Indeed, we show in this report that in obestatin‐treated mice, the type II‐rich skeletal muscle mass increases as well as the size of muscle fibres in the presence of atrophy‐promoting GC, maintaining proteome integrity.

In skeletal muscle, KLF15 is a critical regulator of nutrient catabolism and a key determinant of exercise capacity^7^, ^22^, ^23^; however, its role on atrogene expression is debated. KLF15 is described as a GR target gene acting as a catabolic modulator by regulating the expression of atrogenes under stressful conditions associated with excess levels of GCs.^7^ Paradoxically, GC steroid treatment is used in muscular dystrophy, for example, DMD, where their use exerts ergogenic muscle performance effects.^44^ In *mdx* mice, a genetic model of DMD, the GC treatment improves muscle regeneration and specific force.^45^, ^46^, ^47^ It has been shown that GC steroids rely on KLF15 to mediate ergogenic muscle performance effects. Indeed, deletion of KLF15 in the *mdx* mouse exacerbated the *mdx* muscle phenotype, and muscle‐specific overexpression of KLF15 improved muscle performance. In addition, it appears that low dose and/or transient presence of GC induces KLF15 that controls metabolic genes without inducing atrogenes.^48^ A separate study comparing the effects of daily and weekly administrations of GCs found that KLF15 expression is increased by weekly administration of GCs, but not by chronic administration, which elicited muscle wasting.^49^, ^50^, ^51^ Additionally, intermittent exposure to GCs is associated with epigenetic programming that enhances nutrient utilization by the co‐ordinated action of GR and the transcription factors KLF15 and MEF2C.^52^ Intriguingly, similar effects can be observed in other models of neuromuscular diseases, such as the mouse model of spinal muscular atrophy.^53^ Thus, the regulatory effects of GRs on KLF15 and muscle gene transcription seem to be context specific. An interesting point is the differential effects of GCs, whereby atrophy signalling was induced in healthy animals,^7^, ^54^ while ergogenic effects occur in pathological models, for example, mouse models of DMD, limb‐girdle muscle dystrophy, or spinal muscular atrophy.^48^, ^50^, ^51^, ^52^, ^53^ As such, the down‐regulation of KLF15 leads to decreased expression of atrophy‐related genes. Further studies will be needed to clarify the functional involvement of KLF15 in muscle wasting.

In conclusion, the present study reveals that activation of the obestatin/GPR39 system blocks FoxO action in multiple, and possibly sequential, ways (*Figure* 5D): it promotes post‐translational modifications on the FoxO proteins, including phosphorylation, by Akt and modifies FoxO protein levels by NEDD4‐dependent ubiquitination of KLF15. This dual action of obestatin to trigger FoxO inactivation by obestatin highlights the importance of turning off FoxO in wasting disorder‐related skeletal muscle atrophy, possibly to ensure a tight response to obestatin to counteract catabolic activity. These findings, combined with previous human studies, highlight the potential therapeutic relevance of the obestatin/GPR39 system for fine‐tuning of muscle mass in conditions characterized by muscle atrophy associated with increased circulating GC levels.

## Funding

This work was supported by grants from Instituto de Salud Carlos III in co‐financing with Fondo Europeo de Desarrollo Regional (ISCIII‐FEDER; Ministerio de Ciencia e Innovación (MICINN), Spain; PI17/01707 and PI18/00760), Axencia de Coñecemento en Saúde (ACIS), Servicio Galego de Saúde (SERGAS; Xunta de Galicia; PRIS Program), and Axencia Galega de Innovación (GAIN; Xunta de Galicia; IN607B2019/06).

## Conflict of interest

The authors declare no competing interests.

## Supporting information




**Table S1.** Primary antibodies

## References

[1] Patel R , Williams‐Dautovich J , Cummins CL . Minireview: new molecular mediators of glucocorticoid receptor activity in metabolic tissues. Mol Endocrinol 2014;28:999–1011. 24766141 10.1210/me.2014-1062 PMC5414825

[2] Evans RM . The nuclear receptor superfamily: a rosetta stone for physiology. Mol Endocrinol 2005;19:1429–1438. 15914712 10.1210/me.2005-0046

[3] Meijsing SH , Pufall MA , So AY , Bates DL , Chen L , Yamamoto KR . DNA binding site sequence directs glucocorticoid receptor structure and activity. Science 2009;324:407–410. 19372434 10.1126/science.1164265 PMC2777810

[4] Zierath JR , Wallberg‐Henriksson H . From receptor to effector: insulin signal transduction in skeletal muscle from type II diabetic patients. Ann N Y Acad Sci 2002;967:120–134. 12079842 10.1111/j.1749-6632.2002.tb04270.x

[5] Kuo T , Harris CA , Wang JC . Metabolic functions of glucocorticoid receptor in skeletal muscle. Mol Cell Endocrinol 2013;380:79–88. 23523565 10.1016/j.mce.2013.03.003 PMC4893778

[6] Watson ML , Baehr LM , Reichardt HM , Tuckermann JP , Bodine SC , Furlow JD . A cell‐autonomous role for the glucocorticoid receptor in skeletal muscle atrophy induced by systemic glucocorticoid exposure. Am J Physiol Endocrinol Metab 2012;302:E1210–E1220. 22354783 10.1152/ajpendo.00512.2011 PMC3361985

[7] Shimizu N , Yoshikawa N , Ito N , Maruyama T , Suzuki Y , Takeda S , et al. Crosstalk between glucocorticoid receptor and nutritional sensor mTOR in skeletal muscle. Cell Metab 2011;13:170–182. 21284984 10.1016/j.cmet.2011.01.001

[8] Menconi M , Fareed M , O'Neal P , Poylin V , Wei W , Hasselgren PO . Role of glucocorticoids in the molecular regulation of muscle wasting. Crit Care Med 2007;35:S602–S608. 17713416 10.1097/01.CCM.0000279194.11328.77

[9] Schakman O , Gilson H , Thissen JP . Mechanisms of glucocorticoid‐induced myopathy. J Endocrinol 2008;197:1–10. 18372227 10.1677/JOE-07-0606

[10] Hu Z , Wang H , Lee IH , Du J , Mitch WE . Endogenous glucocorticoids and impaired insulin signaling are both required to stimulate muscle wasting under pathophysiological conditions in mice. J Clin Invest 2009;119:3059–3069. 19759515 10.1172/JCI38770 PMC2752071

[11] Gurriarán‐Rodríguez U , Santos‐Zas I , Al‐Massadi O , Mosteiro CS , Beiroa D , Nogueiras R , et al. The obestatin/GPR39 system is up‐regulated by muscle injury and functions as an autocrine regenerative system. J Biol Chem 2012;287:38379–38389. 22992743 10.1074/jbc.M112.374926 PMC3488106

[12] Gurriarán‐Rodríguez U , Santos‐Zas I , González‐Sánchez J , Beiroa D , Moresi V , Mosteiro CS , et al. Action of obestatin in skeletal muscle repair: stem cell expansion, muscle growth, and microenvironment remodeling. Mol Theraphy 2015;23:1003–1021. 10.1038/mt.2015.40 PMC4817756 25762009

[13] Santos‐Zas I , Gurriarán‐Rodríguez U , Cid‐Díaz T , Figueroa G , González‐Sánchez J , Bouzo‐Lorenzo M , et al. β‐Arrestin scaffolds and signaling elements essential for the obestatin/GPR39 system that determine the myogenic program in human myoblast cells. Cell Mol Life Sci 2016;73:617–635. 26211463 10.1007/s00018-015-1994-z PMC11108386

[14] Santos‐Zas I , Negroni E , Mamchaoui K , Mosteiro CS , Gallego R , Butler‐Browne GS , et al. Obestatin increases the regenerative capacity of human myoblasts transplanted intramuscularly in an immunodeficient mouse model. Mol Ther 2017;25:2345–2359. 28750736 10.1016/j.ymthe.2017.06.022 PMC5628792

[15] González‐Sánchez J , Sánchez‐Temprano A , Cid‐Díaz T , Pabst‐Fernández R , Mosteiro CS , Gallego R , et al. Improvement of Duchenne muscular dystrophy phenotype following obestatin treatment. J Cachexia Sarcopenia Muscle 2018;9:1063–1078. 30216693 10.1002/jcsm.12338 PMC6240759

[16] Cid‐Díaz T , Santos‐Zas I , González‐Sánchez J , Gurriarán‐Rodríguez U , Mosteiro CS , Casabiell X , et al. Obestatin controls the ubiquitin‐proteasome and autophagy‐lysosome systems in glucocorticoid‐induced muscle cell atrophy. J Cachexia Sarcopenia Muscle 2017;8:974–990. 28675664 10.1002/jcsm.12222 PMC5700440

[17] Mamchaoui K , Trollet C , Bigot A , Negroni E , Chaouch S , Wolff A , et al. Immortalized pathological human myoblasts: towards a universal tool for the study of neuromuscular disorders. Skelet Muscle 2011;1:34. 22040608 10.1186/2044-5040-1-34 PMC3235972

[18] Thorley M , Duguez S , Mazza EMC , Valsoni S , Bigot A , Mamchaoui K , et al. Skeletal muscle characteristics are preserved in hTERT/cdk4 human myogenic cell lines. Skelet Muscle 2016;6:43. 27931240 10.1186/s13395-016-0115-5 PMC5146814

[19] Hollenberg SM , Weinberger C , Ong ES , Cerelli G , Oro A , Lebo R , et al. Primary structure and expression of a functional human glucocorticoid receptor cDNA. Nature 1995;318:635–641. 10.1038/318635a0 PMC6165583 2867473

[20] Hinds TD Jr , Ramakrishnan S , Cash HA , Stechschulte LA , Heinrich G , Najjar SM , et al. Discovery of glucocorticoid receptor‐beta in mice with a role in metabolism. Mol Endocrinol 2010;24:1715–1727. 20660300 10.1210/me.2009-0411 PMC2940475

[21] Kino T , Manoli I , Kelkar S , Wang Y , Su YA , Chrousos GP . Glucocorticoid receptor (GR) β has intrinsic, GRα‐independent transcriptional activity. Biochem Biophys Res Commun 2009;381:671–675. 19248771 10.1016/j.bbrc.2009.02.110 PMC2796800

[22] Gray S , Wang B , Orihuela Y , Hong EG , Fisch S , Haldar S , et al. Regulation of gluconeogenesis by Krüppel‐like factor 15. Cell Metab 2007;5:305–312. 17403374 10.1016/j.cmet.2007.03.002 PMC1892530

[23] Haldar SM , Jeyaraj D , Anand P , Zhu H , Lu Y , Prosdocimo DA , et al. Kruppel‐like factor 15 regulates skeletal muscle lipid flux and exercise adaptation. Proc Natl Acad Sci U S A 2012;109:6739–6744. 22493257 10.1073/pnas.1121060109 PMC3340075

[24] Jeyaraj D , Scheer FA , Ripperger JA , Haldar SM , Lu Y , Prosdocimo DA , et al. Klf15 orchestrates circadian nitrogen homeostasis. Cell Metab 2010;15:311–323. 10.1016/j.cmet.2012.01.020 PMC3299986 22405069

[25] Sandri M , Sandri C , Gilbert A , Skurk C , Calabria E , Picard A , et al. Foxo transcription factors induce the atrophy‐related ubiquitin ligase atrogin‐1 and cause skeletal muscle atrophy. Cell 2004;117:399–412. 15109499 10.1016/s0092-8674(04)00400-3 PMC3619734

[26] Waddell DS , Baehr LM , van den Brandt J , Johnsen SA , Reichardt HM , Furlow JD , et al. The glucocorticoid receptor and FOXO1 synergistically activate the skeletal muscle atrophy‐associated MuRF1 gene. Am J Physiol Endocrinol Metab 2008;295:E785–E797. 18612045 10.1152/ajpendo.00646.2007 PMC2652500

[27] Leenders JJ , Wijnen WJ , Hiller M , van der Made I , Lentink V , van Leeuwen RE , et al. Regulation of cardiac gene expression by KLF15, a repressor of myocardin activity. J Biol Chem 2010;285:27449–27456. 20566642 10.1074/jbc.M110.107292 PMC2930743

[28] Lee DS , Choi H , Han BS , Kim WK , Lee SC , Oh KJ , et al. c‐Jun regulates adipocyte differentiation via the KLF15‐mediated mode. Biochem Biophys Res Commun 2016;469:552–558. 26692489 10.1016/j.bbrc.2015.12.035

[29] Hirata Y , Nomura K , Senga Y , Okada Y , Kobayashi K , Okamoto S , et al. Hyperglycemia induces skeletal muscle atrophy via a WWP1/KLF15 axis. JCI Insight 2019;4:e124952. 10.1172/jci.insight.124952 PMC6478420 30830866

[30] Stitt TN , Drujan D , Clarke BA , Panaro F , Timofeyva Y , Kline WO , et al. The IGF‐1/PI3K/Akt pathway prevents expression of muscle atrophy‐induced ubiquitin ligases by inhibiting FOXO transcription factors. Mol Cell 2004;14:395–403. 15125842 10.1016/s1097-2765(04)00211-4

[31] Calnan DR , Brunet A . The FoxO code. Oncogene 2008;27:2276–2288. 18391970 10.1038/onc.2008.21

[32] Tia N , Singh AK , Pandey P , Azad CS , Chaudhary P , Gambhir IS . Role of forkhead box O (FOXO) transcription factor in aging and diseases. Gene 2018;648:97–105. 29428128 10.1016/j.gene.2018.01.051

[33] Koncarevic A , Jackman RW , Kandarian SC . The ubiquitin‐protein ligase Nedd4 targets Notch1 in skeletal muscle and distinguishes the subset of atrophies caused by reduced muscle tension. FASEB J 2007;21:427–437. 17172638 10.1096/fj.06-6665com

[34] Cao XR , Lill NL , Boase N , Shi PP , Croucher DR , Shan H , et al. Nedd4 controls animal growth by regulating IGF‐1 signaling. Sci Signal 2008;1:ra5. 18812566 10.1126/scisignal.1160940 PMC2833362

[35] Liu Y , Oppenheim RW , Sugiura Y , Lin W . Abnormal development of the neuromuscular junction in Nedd4‐deficient mice. Dev Biol 2009;330:153–166. 19345204 10.1016/j.ydbio.2009.03.023 PMC2810636

[36] Fouladkou F , Lu C , Jiang C , Zhou L , She Y , Walls JR , et al. The ubiquitin ligase Nedd4‐1 is required for heart development and is a suppressor of thrombospondin‐1. J Biol Chem 2010;285:6770–6780. 20026598 10.1074/jbc.M109.082347 PMC2825471

[37] Nagpal P , Plant PJ , Correa J , Bain A , Takeda M , Kawabe H , et al. The ubiquitin ligase Nedd4‐1 participates in denervation‐induced skeletal muscle atrophy in mice. PLoS ONE 2012;7:e46427. 23110050 10.1371/journal.pone.0046427 PMC3482220

[38] Huang X , Chen J , Cao W , Yang L , Chen Q , He J , et al. The many substrates and functions of NEDD4. Cell Death Dis 2019;10:904. 31787758 10.1038/s41419-019-2142-8 PMC6885513

[39] Zhao J , Brault JJ , Schild A , Cao P , Sandri M , Schiaffino S , et al. FoxO3 coordinately activates protein degradation by the autophagic/lysosomal and proteasomal pathways in atrophying muscle cells. Cell Metab 2007;6:472–483. 18054316 10.1016/j.cmet.2007.11.004

[40] Webb AE , Brunet A . FOXO transcription factors: key regulators of cellular quality control. Trends Biochem Sci 2014;39:159–169. 24630600 10.1016/j.tibs.2014.02.003 PMC4021867

[41] Milan G , Romanello V , Pescatore F , Armani A , Paik JH , Frasson L , et al. Regulation of autophagy and the ubiquitin‐proteasome system by the FoxO transcriptional network during muscle atrophy. Nat Commun 2015;6:6670. 25858807 10.1038/ncomms7670 PMC4403316

[42] Cheng Z . The FoxO‐autophagy axis in health and disease. Trends Endocrinol Metab 2019;30:658–667. 31443842 10.1016/j.tem.2019.07.009

[43] Laplante M , Sabatini DM . mTOR signaling in growth control and disease. Cell 2012;149:274–293. 22500797 10.1016/j.cell.2012.03.017 PMC3331679

[44] McDonald CM , Henricson EK , Abresch RT , Duong T , Joyce NC , Hu F , et al. Long‐term effects of glucocorticoids on function, quality of life, and survival in patients with Duchenne muscular dystrophy: a prospective cohort study. Lancet 2018;391:451–461. 29174484 10.1016/S0140-6736(17)32160-8

[45] Anderson JE , McIntosh LM , Poettcker R . Deflazacort but not prednisone improves both muscle repair and fiber growth in diaphragm and limb muscle in vivo in the mdx dystrophic mouse. Muscle Nerve 1996;19:1576–1585. 8941272 10.1002/(SICI)1097-4598(199612)19:12<1576::AID-MUS7>3.0.CO;2-7

[46] St‐Pierre SJ , Chakkalakal JV , Kolodziejczyk SM , Knudson JC , Jasmin BJ , Megeney LA . Glucocorticoid treatment alleviates dystrophic myofiber pathology by activation of the calcineurin/NF‐AT pathway. FASEB J 2004;18:1937–1939. 15456738 10.1096/fj.04-1859fje

[47] Baltgalvis KA , Call JA , Nikas JB , Lowe DA . Effects of prednisolone on skeletal muscle contractility in mdx mice. Muscle Nerve 2009;40:443–454. 19618428 10.1002/mus.21327 PMC2879072

[48] Morrison‐Nozik A , Anand P , Zhu H , Duan Q , Sabeh M , Prosdocimo DA , et al. Glucocorticoids enhance muscle endurance and ameliorate Duchenne muscular dystrophy through a defined metabolic program. Proc Natl Acad Sci U S A 2015;112:E6780–E6789. 26598680 10.1073/pnas.1512968112 PMC4679037

[49] Guerron AD , Rawat R , Sali A , Spurney CF , Pistilli E , Cha HJ , et al. Functional and molecular effects of arginine butyrate and prednisone on muscle and heart in the mdx mouse model of Duchenne Muscular Dystrophy. PLoS ONE 2010;5:e11220. 20574530 10.1371/journal.pone.0011220 PMC2888587

[50] Quattrocelli M , Barefield DY , Warner JL , Vo AH , Hadhazy M , Earley JU , et al. Intermittent glucocorticoid steroid dosing enhances muscle repair without eliciting muscle atrophy. J Clin Invest 2017a;127:2418–2432. 28481224 10.1172/JCI91445 PMC5451235

[51] Quattrocelli M , Salamone IM , Page PG , Warner JL , Demonbreun AR , McNally EM . Intermittent glucocorticoid dosing improves muscle repair and function in mice with limb‐girdle muscular dystrophy. Am J Pathol 2017b;187:2520–2535. 28823869 10.1016/j.ajpath.2017.07.017 PMC5809598

[52] Quattrocelli M , Zelikovich AS , Jiang Z , Peek CB , Demonbreun AR , Kuntz NL , et al. Pulsed glucocorticoids enhance dystrophic muscle performance through epigenetic‐metabolic reprogramming. JCI Insight 2019;4:e132402. 10.1172/jci.insight.132402 PMC6975267 31852847

[53] Walter LM , Deguise MO , Meijboom KE , Betts CA , Ahlskog N , van Westering TLE , et al. Interventions targeting glucocorticoid‐Krüppel‐like factor 15‐branched‐chain amino acid signaling improve disease phenotypes in spinal muscular atrophy mice. EBioMedicine 2018;31:226–242. 29735415 10.1016/j.ebiom.2018.04.024 PMC6013932

[54] van Raalte DH , Ouwens DM , Diamant M . Novel insights into glucocorticoid‐mediated diabetogenic effects: towards expansion of therapeutic options? Eur J Clin Invest 2009;39:81–93. 19200161 10.1111/j.1365-2362.2008.02067.x

[55] von Haehling S , Morley JE , Coats AJS , Anker SD . Ethical guidelines for publishing in the Journal of Cachexia, Sarcopenia and Muscle: update 2019. J Cachexia Sarcopenia Muscle 2019;10:1143–1145. 31661195 10.1002/jcsm.12501 PMC6818444

