# Supplementary material for: Obestatin signalling counteracts glucocorticoid‐induced skeletal muscle atrophy via NEDD4/KLF15 axis
Source: J Cachexia Sarcopenia Muscle. 2021 Mar 9;12(2):493–505. doi: 10.1002/jcsm.12677 (PMC8061369; doi:10.1002/jcsm.12677)
Supplement: Supplementary file 1 — Table S1. Primary antibodies [file JCSM-12-493-s001.docx]

Table S1. Primary antibodies

| Primary antibody | Use | Dilution | Supplier | Reference |
| --- | --- | --- | --- | --- |
| AMPK | WB | 1:1000 | Cell singaling | 2603 |
| Akt | WB | 1:1000 | Cell Signaling | 9272 |
| c-jun | WB | 1:1000 | Cell singaling | 9165 |
| Cathepsin L | WB | 1:1000 | Abcam | ab133641 |
| 4E-BP1 | WB | 1:1000 | Cell Signaling | 9452 |
| ERK1/2 | WB | 1:1000 | Cell singaling | 9102 |
| FoxO1 | WB | 1:1000 | Cell Signaling | 2880 |
| FoxO3a | WB | 1:1000 | Cell Signaling | 12829 |
| FoxO4 | WB | 1:1000 | Cell Signaling | 9472 |
| GAPDH | WB | 1:1000 | Abcam | Ab9485 |
| Glucocorticoid receptors | WB | 1:1000 | Santa Cruz | sc-393232 |
| HA | IF | 2ug/mL | Merck/Sigma | H9658 |
| KLF15 | WB | 1:250 | Santa Cruz | sc-271675 |
| KLF15 | IP | 1:100 | Santa Cruz | sc-271675 |
| LC3A/B | WB | 1:1000 | Cell Signaling | 12741 |
| MAFbx | WB | 1:1000 | Santa Cruz | sc-166806 |
| Murf1 | WB | 1:1000 | Santa Cruz | sc-32920 |
| MHC type I | IF | 2ug/mL | DSHB | BA-F8 |
| MHC type IIa | IF | 2ug/mL | DSHB | SC-71 |
| MHC type IIb | IF | 2ug/mL | DSHB | BF-F3 |
| MHC type IIx | IF | 2ug/mL | DSHB | 6H1 |
| Laminin | **IF** | **1:200** | **Dako/Agilent** | **Z0097** |
| NEDD4 | WB | 1:250 | Santa Cruz | sc-271675 |
| p38 | WB | 1:1000 | Cell Signaling | 9212 |
| p62 | WB | 1:1000 | Cell Signaling | 5114S |
| pAMPK (T172) | WB | 1:1000 | Cell singaling | 2535 |
| pAkt(S473) | WB | 1:1000 | Cell Signaling | 9275 |
| pAkt(S473) | **IF** | **1.200** | **Cell Signaling** | **9275** |
| pp38(T180/Y182) | WB | 1:1000 | Cell Signaling | 4511 |
| p4E-BP1(T37/46) | WB | 1:1000 | Cell Signaling | 9459 |
| pERK1/2(T202/Y204) | WB | 1:1000 | Cell Signaling | 4370 |
| pERK1/2(T202/Y204) | IF | 1:200 | Cell Signaling | 4370 |
| pFoxO1 (Thr24)/FoxO3a (Thr32)/FoxO4 (Thr28) | WB | 1:1000 | Cell Signaling | 2599 |
| pc-jun(S63) | WB | 1:1000 | Cell Signaling | 9261 |
| pS6(S240/244) | WB | 1:1000 | Cell Signaling | 2215 |
| S6 | WB | 1:1000 | Cell Signaling | 2217 |
| Ubiquitin | WB | 1:1000 | Santa Cruz | sc-8017 |
| WWP1 | WB | 1:500 | Proteintech | 13587-1-AP |

Note: Relation of the primary antibodies used in the different analyses performed in this work. WB, western blot; IF, immunofluorescence; IP, co-immunoprecipitation.
